# Metabolomic Profiling of Oral Potentially Malignant Disorders and Its Clinical Values

**DOI:** 10.3390/biomedicines12122899

**Published:** 2024-12-19

**Authors:** Nur Fatinazwa Mohd Faizal, Vui King Vincent-Chong, Anand Ramanathan, Ian C. Paterson, Lee Peng Karen-Ng, Zuraiza Mohamad Zaini

**Affiliations:** 1 Department of Oral and Maxillofacial Clinical Sciences, Faculty of Dentistry, Universiti Malaya, Kuala Lumpur 50603, Malaysia; ftnzwatwork@gmail.com (N.F.M.F.); drranand@um.edu.my (A.R.); 2 Department of Oral Oncology, Roswell Park Comprehensive Cancer Center, Buffalo, NY 14263, USA; vincentvuiking.chong@roswellpark.org; 3 Oral Cancer Research and Coordinating Centre (OCRCC), Faculty of Dentistry, Universiti Malaya, Kuala Lumpur 50603, Malaysia; ipaterson@um.edu.my; 4 Department of Oral and Craniofacial Sciences, Faculty of Dentistry, Universiti Malaya, Kuala Lumpur 50603, Malaysia

**Keywords:** oral potentially malignant disorder, biomarkers, oral squamous cell carcinoma, metabolomics

## Abstract

Oral potentially malignant disorders (OPMD) are a group of lesions carrying the risk of developing into cancer. The gold standard to predict which lesions are more likely to undergo malignant transformation is the presence of dysplasia histologically. However, not all dysplastic lesions progress, and non-dysplastic lesions may also undergo malignant transformation. Oral carcinogenesis is a complex molecular process that involves somatic alterations and the deregulation of transcriptions, protein expression, and metabolite levels. Metabolomics, which is the scientific study of metabolites, has emerged as a promising high-throughput approach to investigate the metabolic changes of small molecules in biological pathways. In this review, we summarize the data relating to the metabolomic profiling of OPMDs, which will help elucidate the complex process of oral carcinogenesis. Furthermore, we identify that among all metabolites, citrate, pyruvate, and glutamate may serve as potential biomarkers for oral leukoplakia (OLK). Notably, metformin and gluconate have been shown to target glutamate and citrate, respectively, in cancer cells. Based on these findings, we propose that targeting these metabolites in patients with OPMD could be a promising therapeutic strategy to mitigate OPMD progression and potentially reduce the risk of malignant transformation. We also discuss the limitations and future directions of metabolomics in OPMD. Understanding these important metabolites is crucial for early detection and monitoring of oral cancer progression.

## 1. Introduction

Oral mucosal lesions carrying the potential to progress into cancer are grouped together as oral potentially malignant disorders (OPMD). These include oral leukoplakia (OLK), oral erythroplakia (OE), proliferative verrucous leukoplakia (PVL), oral submucous fibrosis (OSMF), oral lichen planus (OLP), and oral lichenoid lesions (OLL). The overall prevalence and malignant transformation (MT) rate of OPMD are 4.5% and 7.9%, respectively [1]. Among OPMDs, leukoplakia is the most common lesion, with a MT rate of 9.8% over a 5-year period (2015–2020) [2].

The current standard of practice for detecting oral epithelial dysplasia (OED) is histopathological examination through biopsy tissues. The grading of dysplasia, which is categorized into mild, moderate, and severe, has been widely used to assess the risk of OED progressing into cancer. However, the OED grading has limitations in clinical settings and is also proven to be a poor predictor of malignant transformation due to significant inter-observer and intra-observer variability in several large studies [3,4]. In addition, some OPMDs do not transform into cancer as they may regress, and even non-dysplastic lesions may develop into cancer [5,6]. As a result, accurately distinguishing between high-risk and low-risk lesions remains challenging, rendering current standards for the management and diagnosis of suboptimal OPMDs [7].

Both dysplastic and non-dysplastic OPMDs exhibit various genetic alterations. These alterations involve the inactivation of tumor suppressor genes through mutations or deletions, as well as the activation of proto-oncogenes via mutations, gene amplification, or chromosomal translocations [8]. The accumulation of these genetic changes is thought to contribute to the dysregulation of cellular processes, such as cell differentiation, cell division, cell proliferation, apoptosis, DNA repair, and immune response [9,10]. Frequent loss of heterozygosity at 3p, 9p, and 17p in dysplasia suggests these are early cytogenetic changes in oral carcinogenesis, while alterations in 11q, 4q, and chromosome 8 appear to be later events in the development of oral squamous cell carcinoma (OSCC) [11].

Metabolomics, the scientific study of metabolites, has emerged as a promising approach to investigate the metabolic changes associated with various diseases, including cancer [12,13,14,15]. Metabolites are small molecules found within biofluids, cells, organisms, and tissues, and their profiling can provide valuable insights into the metabolic alterations occurring in disease states. In recent years, there has been growing evidence of the utility of metabolomic techniques in studying the metabolite signatures of OPMD and OSCC. These metabolomic studies aim to identify specific metabolites or metabolic pathways with the potential to serve as biomarkers for early detection or prognostic indicators of OPMD and OSCC.

In this review, we summarize findings from metabolomic studies on OPMDs, and in particular oral leukoplakia, which has the highest rate of MT. We also discuss the importance of these metabolites in revealing metabolic biomarker signatures, which might serve as adjunct tools for diagnosis and prognosis. Additionally, we highlight specific metabolites and their potential applications for the management of OPMDs. Some future directions and opportunities for metabolomic studies in OPMDs are also discussed.

## 2. Oral Potentially Malignant Disorders (OPMD)

Numerous studies have reported that OSCC is frequently preceded by OPMDs [1,6,16,17]. The World Health Organization (WHO) refers to the term “oral potentially malignant disorder” (OPMD) as oral lesions and conditions with an increased risk of malignant transformation [16]. These disorders encompass a range of diseases with diverse clinical presentations, histological subtypes, and underlying risk factors or etiologies. This includes leukoplakia, erythroplakia, erythroleukoplakia, oral lichen planus, oral submucous fibrosis (OSF), and oral dysplasia. The clinical features of these OPMDs are described in Table 1.

## 3. Metabolomic Profiling in OPMD

### 3.1. Metabolomic Techniques Used for OPMD

There are two widely used techniques adopted in metabolic studies, namely nuclear magnetic resonance spectroscopy (NMR) and mass spectrometry (MS). These two techniques are either used alone or together with other separation techniques, including liquid chromatography (LC), gas chromatography (GC), and capillary electrophoresis (CE). NMR provides outstanding analytical precision in the identification of cancer metabolomic signature [10] but despite yielding exceedingly reproducible and accurately quantified data, this technique has low sensitivity [12,18].

In contrast to NMR, MS displays high sensitivity and wider metabolome coverage. This approach ionizes chemical species and sorts the ions based on their mass-to-charge ratio, forming a mass spectrum. Mass spectrometry is usually used together with chromatography and other separation techniques to ameliorate the capability of mass resolving and mass determining. LC-MS is known as the most adaptable MS-based technique that is suitable to evaluate a wide variation of compounds due to numerous types of mass spectrometers and mobile and stationary phases used. GC-MS, on the other hand, is suitable for assessing metabolites with low molecular weight but is restricted to volatile compounds or can be made volatile by derivatization [12,18,19]; CE-MS is suitable to analyze polar or ionic compounds but has lower sensitivity and reproducibility in comparison to LC-MS and GC-MS [12,20,21]. However, these platforms are not able to capture all metabolites in a single experiment [19,22].

### 3.2. Biological Samples Used for OPMD Metabolomic

**Figure 1:**
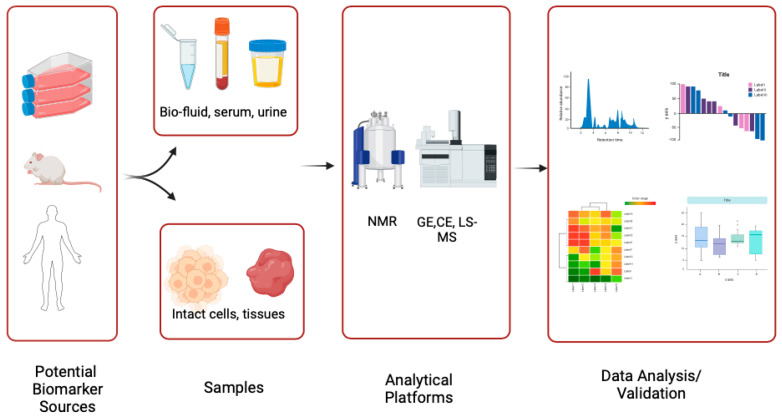
Metabolomic profiling workflow from sample collection through to data analysis, illustrating each stage from sample preparation, metabolite extraction, and detection to data processing and interpretation.

Many metabolomics-based studies have reported that thousands of metabolites can be detected in biofluids such as saliva, serum, plasma, and urine to explore the metabolomic signatures [18]. Furthermore, cell and tissue extracts are also widely used in metabolomic profiling studies [23]. The process of metabolomic analysis in OPMD and OSCC is depicted in Figure 1.

#### 3.2.1. Salivary Metabolomics

There is a growing interest in utilizing bio-fluids such as saliva to investigate oral cancer-associated biomarkers; saliva is easily collected, and sampling is non-invasive [23,24]. For metabolomic studies, saliva is collected using either stimulated or unstimulated methods, which result in a different chemical composition. Therefore, it is crucial to identify the type of saliva to be used [25]. Studies have reported that salivary metabolic profiles reflect the pathological and physiological states, thus furnishing valuable information about systemic or localized health states [12,26,27]. Many factors can influence the salivary metabolome, including the method, collection time, overall oral health status, residual food, food intake, as well as the oral microbiome [12]. Salivary and tissue metabolomic studies have revealed consistent alterations in the levels of various metabolites in oral cancer compared to control samples. This suggests that salivary metabolomics could provide valuable insights into the molecular mechanisms underlying oral carcinogenesis.

#### 3.2.2. Serum and Plasma Metabolomics

In addition to saliva, serum and plasma are also commonly used for metabolomic-based studies [28,29]. Serum is a non-cellular, protein-rich fluid obtained following blood coagulation, and plasma is a cellular component containing white blood cells, red blood cells, and platelets [29]. Both components contain a broad array of metabolites, which can be influenced by various preanalytical factors such as the type of tube collection used [12].

It has been reported that abnormal metabolic activity in serum results in alterations of several metabolites, including amino acids, glucose, pyruvate, ketone bodies, and lactate, which implicates many biological pathways such as amino acid catabolism, altered lipolysis, and the TCA cycle [30].

#### 3.2.3. Cell and Tissue Metabolomics

The gold standard for predicting cancer development from OPMDs remains incisional biopsy and subsequent histopathological interpretation. However, this procedure is inherently subjective and often fails to capture the full spectrum of heterogeneity in neoplastic processes. Consequently, distinguishing between non-progressing and progressing precancerous lesions remains a significant challenge [31,32].

Most metabolomics research relies on biofluids due to their minimally invasive sampling methods. However, it is not organ-specific and reflects a broad spectrum of biochemical processes occurring throughout the body [33]. While biofluids offer valuable insights into OSCC pathogenesis and may show similar findings as those obtained from tissue analyses, some pathophysiological mechanisms related to tumor development and progression can generate compounds that can be more effectively detected directly on the affected site [34]. Additionally, the comparison between cancerous and normal tissues, as well as different zones within the same tumor specimen, can be reliably conducted using tissue samples. Hence, there has been increased interest in using tissue samples for metabolomic investigations of OSCC. Metabolic alterations have been extensively studied in OSCC derivatives, including cell lines and animal models, to explore their implication in OSCC pathogenesis, cell death, drug resistance, and metastasis potential [35,36,37,38,39].

#### 3.2.4. Urinary Metabolomics

Urine samples are also used in metabolomic studies; however, such samples are not common in oral cancer research. Urine is broadly used in other metabolomic studies as it is easy to collect and possibly contains a broad range of metabolites [28]. To date, only one study has reported the use of urine samples in metabolomic studies of oral cancer [40].

## 4. Metabolites Associated with OPMD

### 4.1. Oral Leukoplakia (OLK)

The World Health Organization (WHO) has recognized oral leukoplakia (OLK), erythroplakia, erythroleukoplakia, oral lichen planus (OLP), and oral submucous fibrosis (OSF) as a part of OPMD. To date, metabolic profiling using different types of samples has only been conducted in OLK, OLP, and OSF and their differences compared with OSCC (Table 2 and Table 3). However, a direct comparison of metabolomic profiles may be difficult, as some studies have considered OLK and OLP as a single group of OPMD [41].

A few studies have used salivary metabolomics to discriminate OLK from OSCC [42,43]. The first study was performed by Yan and colleagues, who reported that salivary metabolic profiles obtained using high-performance liquid chromatography mass spectrometry (HPLC-MS) could precisely diagnose and distinguish OLK and OSCC [44]. The specific metabolites were not disclosed; however, it was reported that there was a clear distinction between OSCC, OPMD, and the healthy controls. A similar study by Wei et al. performed salivary metabolomic profiling on OSCC, OLK, and a healthy control group using ultraperformance liquid chromatography coupled with quadrupole/time-of-flight mass spectrometry (UPLC-QTOF-MS). The results showed that 41 discriminant metabolites were identified between OSCC and the controls, 61 between OSCC and OLK, and 27 between OLK and the controls. A panel of five of the most discriminatory metabolites was g-aminobutyric acid (GABA), valine, phenylalanine, lactic acid, and n-eicosanoic acid. It was also found that the combination of valine, phenylalanine, and lactic acid represented a powerful predictive biomarker in discriminating OSCC from the control and OLK tissues [43]. More recently, Sridharan and others demonstrated a range of salivary metabolites that were significantly altered in OLK and OSCC using a similar analytical platform. A total of 31 salivary metabolites were upregulated, including 2-oxoarginine, 1-methylhistidine, d-glycerate-2-phosphate, inositol 1,3,4-triphosphate, 4-nitoquinolone–oxide sphinganine-1-phosphate, norcocaine nitroxide, and pseudouridine, and 11 metabolites were downregulated, including L-homocysteic acid, estradiol valerate, and neuraminic, in OSCC and OLK [42]. Kitabatake et al. (2023) used CE-MS to discriminate OLK with and without dysplasia. It was reported that three metabolites, guanine, carnitine, and N-acetylputrescine, were able to differentiate between OLK and the healthy controls, and only 7-methylgyanine could differentiate dysplastic OLK and non-dysplastic OLK [45]. A further 15 significantly different compounds were also found using GC-MS to distinguish OLK from OSCC [15].

Serum metabolomic profiling performed using ^1^H NMR was also reported to discriminate between OSCC, OLK, and the healthy controls [46,47]. Gupta and colleagues found that a combination of discriminant metabolites consisting of glutamine, acetate, acetone, and choline was able to precisely discriminate OLK from OSCC. Meanwhile, glutamine, acetone, choline, and propionate alone were able to distinguish OSCC from the healthy controls [46]. These alterations were suggested to contribute to the development of oral cancer, thus suggesting that plasma metabolites may serve as a potential metabolic biomarker for oral cancer progression.

A urinary metabolomic study was also performed using GC-MS to discriminate between OLK and OSCC patients. A panel of differentially expressed metabolites was identified, whereby a combination of 6-hydroxynicotinic acid, tyrosine, and cysteine yielded high accuracy, sensitivity, and specificity at 92.7%, 85%, and 89.7%, respectively, in discriminating OLK from OSCC. This metabolite combination also demonstrated 91.9% positive predictive value [40]. Intriguingly, these studies reported that valine levels were decreased in both the saliva and serum of OSCC patients compared to OLK. Valine is a branched-chain amino acid, the catabolic process of which is known to be essential for cancer cells. A decrease in valine levels in OSCC in comparison to OPMD and the healthy controls may be due to its association with amino acid catabolism and increased metabolic utilization during oral carcinogenesis [37,43,46]. It was also identified that lactic acid and choline levels increased in OSCC in relation to subjects with OLK [37,46]. This could be due to the role of amino acids in energy production and the growth of cancer cells, respectively [24].

Cell metabolomic analysis was also performed using GC-MS to distinguish between OED, OSCC (BICR3), and normal human oral keratinocytes (NHOK810) cell lines. This study investigated potential metabolites that could differentiate between high-risk OED, low-risk OED, and normal OED. High-risk OED refers to the dysplastic cell lines with a high risk of transforming into cancer (D4, D9, D19, D20, D34, D35, and DOK), whereas low-risk OED refers to the dysplastic cell lines with a low risk of transforming into cancer (D6, D25, D30, E4, and D17) (as previously described in [48]). Several metabolites were found to be elevated in high-risk OED compared to low-risk OED, including those involved in amino acid, pyrimidine, and sterol metabolism. Out of the many metabolites detected, citrate was confirmed by targeted metabolomics to be elevated in OLK compared in relation to other groups. The level of citrate was consistently elevated in the conditioned media of high-risk OED cell lines D4, D19, D35, and D17, relative to the normal human oral keratinocytes and low-risk OED cell lines D6 and D30 [48]. All cell lines were derived from patients who were clinically diagnosed with leukoplakia, except D19 and D35, which were erythroleukoplakia. Pyruvate, glutamine, lysine, and methionine were also reported to critically distinguish oral precancerous cells (DOK) from normal human oral epithelial cells and oral cancer cells using GC-MS [49].

### 4.2. Oral Lichen Planus (OLP)

Several studies have also focused on the metabolic profiling of OLP. Similar to OLK, Yan et al. were the first group to propose a metabolomic-based diagnostic approach to discriminate OLP from OSCC using salivary metabolites [43]. They reported that 14 OLP-related biomarkers were discovered by high-performance liquid chromatography mass spectrometry (HPLC/MS) analysis but were not revealed. Ishikawa et al. reported 14 metabolites were found to be significantly higher in OSCC than OLP groups using CE-TOF-MS analysis [50]. This was the first study to reveal that a combination of two metabolites, indole-3-acetate and ethanolamine phosphate, exhibited a high AUC for accurately distinguishing OSCC from OLP using multiple logistic regression (MLR). Indole -3-acetate promotes the growth of cancer cells and their adjacent cells, which become malignant, whereas ethanolamine phosphate is an intermediate metabolite of sphingosine -1-phosphate (S1P), which is increased in malignant tumors and involved in tumor progression [51]. These two metabolites have been reported to be increased in other cancers but not in OSCC. This suggests that the combination of these salivary metabolites may provide insightful knowledge for clinicians and specialists in detecting potential oral lesions that could progress to malignancy [50].

Prior to this study [50], salivary biomarkers such as 8-hydroxy-2-deoxyguanosine (8-OHdG), malondialdehyde (MDA), vitamin C, and vitamin E have been measured between OSCC, OPMD consisting of OLK, OLP, OSF, and healthy controls. The results demonstrated that 8-OHdG and MDA levels, measured by ELISA and thiobarbituric acid (TBA) reaction, respectively, were significantly higher in OSCC and OPMD compared to the controls. In contrast, the levels of vitamin C and vitamin E, measured by HPLC, were higher in the controls compared to OSCC and OPMD patients [47]. This suggests lipid damage and oxidative DNA are present in OPMD and oral cancer patients; thus, these salivary biomarkers could contribute to the diagnosis of the diseases [52].

Wang and others demonstrated three prominent metabolites for the early detection of OLP, which are glutamic acid, lysoPE (18:0), and taurine, performed using ultra-performance liquid chromatography-quadrupole/Orbitrap high-resolution mass spectrometry (UHPLC-Q-Orbitrap HRMS). This panel was selected due to its high AUC, specificity, and sensitivity, at 0.938, 84.4%, and 90.5%, respectively [53]. Similarly, Li X et al. (2022) also identified uridine, taurine, citric acid, glutamate, and LysoPC (18:1) metabolites as general biomarkers for OLP malignant transformation [54]. Another study also identified 14 differential metabolites between the two most common types of OLP, reticulated type (ROLP) and erosion type (EOLP), including glutamic acid, tryptophan, indoleacrylic acid, eicosapentaenoic acid, citric acid, and benzamide [55]. The correlation of these shared metabolites was analyzed, and six important metabolic pathways were determined. This shows that the metabolic changes were closely correlated with abnormal amino acids in OLP patients. Ten potential biomarkers were also reported by Yang X. et al. (2017) in serum samples between OELP and control patients [56]. Yang et al. [57] identified 21 modulated metabolites using a liquid chromatography (LC-mass spectrometry (MS)) system (LC-MS), of which 13 of them were upregulated and 8 were downregulated in the reticular OLP group compared to the control group. Subsequently, the authors also discovered 30 differentially expressed metabolites using LC-MS on urinary samples, in which 13 metabolites were upregulated and 17 metabolites were downregulated in the reticular OLP group compared to the control group [57,58].

### 4.3. Oral Submucous Fibrosis (OSF)

Goel et al. identified the amino acid profile in OSF plasma samples using high-performance liquid chromatographic analysis (HPLC). Their findings reported a decrease in the assay levels of arginine, histidine, isoleucine, leucine, threonine, and tyrosine in all of the OSF groups. Increased levels of alanine and methionine were observed in all of the OSF groups as well. However, levels of glycine and serine were found to decline in mild and severe OSF groups but escalated in the moderate group. Whereas the assay levels of valine, lysine, and phenylalanine were observed to decline in moderate and severe groups but increased in the mild group. This suggests the amino acid profiles progressively decrease with disease severity, which may be due to their increased usage in the synthesis of collagen and cross linking [59].

To our knowledge, only one study has investigated the discrimination of OSF from the OSCC and healthy control groups with tissue samples. Musharraf and others used gas chromatography coupled with triple quadrupole tandem mass spectrometry (GS-MS) and found 31 out of 735 compounds could distinguish between the OSCC, OPMD, and control samples, of which 19 of them were identified metabolites. Among these metabolites, nine of them were amino acids that showed gradual amino acid concentration decreases from the healthy control to OSF and to OSCC tissues, including alanine, glutamine, glycine, glutamic acid, lysine, norleucine, serine, and threonine. It was suggested that the decreased levels of these amino acids were due to the enhanced metabolic utilization or biosynthetic pathways upregulation, which are needed in cell proliferation in cancer [60]. The modulation of amino acid metabolism could serve as a potential strategy by which to detect OPMD and oral cancer.

The present review on different metabolomic research in OPMD shows that over 100 discriminant metabolites of different types can be demonstrated. Table 3 delineates some of the significant metabolite markers between OPMD and OSCC and normal samples.

**Table 2:** Oral potentially malignant disorder (OPMD)-associated biomarkers proposed by oral metabolomic studies based on nuclear magnetic resonance spectroscopy (NMR) and mass spectrometry (MS) approaches.

OPMD	Sample Type	Analytical Platform	Metabolites	Pathway	References
OLK	Saliva	UPLC-QTOF-MS	GABA, phenylalanine, valine, *n*-eicosanoic acid, lactate, alanine, isoleucine, leucine, *n*-Tetreadecanoic acid, proline, threonine, *n*-Dodecanoic acid, 3-Indolepropionic acid, *n*-Ecicosanoic acid, Homocysteine, 4, Methoxyhenylacetic acid	Glycolysis, amino acid, fatty acid	(Wei et al., 2011) [43]
OLK, OLP	Saliva	HPLC/MS	Not disclosed	Not disclosed	(Yan et al., 2008) [44]
OLK	Saliva	QTOF-MS	1-methylhistidine, inositol 1,3,4-triphosphate, d-glycerate-2-phosphate, 4-nitroquinoline-1-oxide, 2-oxoarginine, norcocaine nitroxide, sphinganine-1-phosphate, pseudouridine, L-homocysteic acid ubiquinone, neraminic acid, estradiol valerate	Sphingolipid, amino acid, carbohydrate, oxidative stress, estrogen, nucleotide biosynthesis, electron transport chain	(Srindharan et al., 2018) [61]
OLK	Saliva	CE-MS	Guanine, carnitine, N-acetylputrescine, 7-metylguanine	Arginine decarboxylase pahway, fatty acid	(Kitabatake K et al., 2023) [45]
OLK	Saliva	GC-MS	Decanedioic acid, 2-methyloctacosane, eicosane, octane, 3,5-dimethyl, pentadecane, hentriacontane, 5, 5-diethylpentadecane, nonadecane, oxalic acid, 6-phenylundecanea, l-proline, 2-furancarboxamide, 2-isopropyl-5-methyl-1-heptanol, pentanoic acid, Docosane.		(Tantray S et al., 2022) [15]
OLK	Serum	^1^H NMR	Glutamine, acetone, acetate, choline, propionate, threonine	Amino acid, choline, ketone body	(Gupta et al., 2015) [46]
OLK	Urine	GC-MS	Valine, 6-hydroxynicotic acid, cysteine, tyrosine, alanine, leucine, cystine, serine, Hippurate, phenylalanine, histamine, tryptophan	Glycolysis, amino acid,	(Xie et al., 2012) [40]
OLK, ELK	Cell line	GC-MS	Citrate	TCA	(Karen-Ng et al., 2021) [48]
OLP	Saliva (UW)	CE-MS	Ornithine, carnitine arginine, o-hydroxybenzoate, N-acetylglucosamine-1-phosphate, ribose-5-phosphate	Pentose phosphate, urea cycle	(Ishikawa et al., 2018) [62]
OLP	Saliva (UW)	CE-MS	Indole-3-acetate, ethanolamine phosphate, trimethylamine N-oxide, putrescine, creatinine, 5-aminovaerate, pipecolate, N-acetylputerscine, gamma-Butyrobetaine, N-acetylhistidine, o-Acetylcartinine, N_1_-Acetylspermine, 2^1^- Deoxyinosine, N-Acetylglucosamine	Lysine metabolism, sphingolipid, amino acid	(Ishikawa et al., 2019) [63]
OLP	Saliva (UW)	CPSI-MS	Putrescine, cadaverine, thymidine, adenosine, 5-aminopentoate, Hippuric acid, phosphocholine, glucose, serine, adrenic acid	aminoacyl tRNA biosynthesis, arginine/proline metabolism arginine biosynthesis lysine degradation and histidine metabolism	(Song et al., 2020) [41]
OLP	Serum	UHPLC/Q-Orbitrap HRMS	Glutamic acid, LysoPC(18:0), taurine	Taurine and hypotaurine metabolism, amino acid metabolism	(Wang XS et al., 2020) [53]
OLP	Serum	UHPLC/Q-Orbitrap HRMS	12 differential metabolites between OELP and ROLP	Alanine. Aspartate, glutamate matabolism	(Xin MZ et al., 2021) [55]
OLP	Serum	UPLC-QTOF- MS	10 differential metabolites between OELP and Control	Receptor-mediated G-protein linked signaling pathway, glycosylphosphatidylinositol-anchor biosyntehesis, glycerophospholipid metabolism	(Yang X et al., 2017) [56]
OLP	Plasma	UHPLC/Q-Orbitrap HRMS	Sphingosine, deoxycholic, 3b,7a-Dihydroxy-5b-chlanoic acid, 1-linoleoyl-sn-glycero-3-phosphoethanolamine,citric acid, isoleucine, cis-5-tetradecanolycarnitinem decanoyl carnitine, lactic acid, docosahexaenoic acid, 2-amino- 1,3,4-octadecanetriol, arginine, acetyl carnitine	Primary bile acid biosynthesis, alanine. Aspartate, glutamate metabolism	(Li X et al., 2022) [64]
OLP	Urine	UHPLC/Q-Orbitrap HRMS	6-methyladenine, phenylpyruvic acid, ribonic acid, L-arginine, Gamma-glutamyltyrosine, kynurenic acid, phytosphingosine, L-gamma-glutamyl-L-isoleucine, sphinganine, panthetheine, L-urobillin, L-urobillinogen	Sphingolipid metabolism, porphyrin and chlorophyll metabolism, arginine/proline metabolism, tryptophan metabolism.	(Li XZ et al., 2017) [54]
OLP	Urine	LC-MS	30 differentially expressed metabolites identified of reticular OLP	Carbohydrate metabolism	(Yang et al., 2020) [58]
OLP	Tissue	LC-MS	p-chlorophenylalanine, 6,8-Dihydroxypurine, taurine, malic acid, L-Glutamic acid, choline, adrenochrome, lactic acid, aspartaic acid, L-acetylcarnitine, L-serine,didymin, L-glutamine, S-ethyl isothiourea, PE(P-18:1(9Z)/16:1(9Z)), threonic acid, L-histidine, L-tryptophan, prostaglandin E2, guanine, citric acid	Aminoacyl-tRNA biosynthesis, alanine, aspartate, and glutamate metabolism, D-glutamine and D-glutamate metabolism, Glycine, serine, and threonine metabolism, Histidine metabolism, Pyruate metabolism, Taurine and hypotaurine metabolism, Tryptophan metabolism	(Yang X.Y et al., 2018) [57]
OSF	Tissue	GC-MS	31 differential compounds (19 identified and 12 unidentified	Not disclosed	(Musharraf et al., 2016) [60]

OLK, oral leukoplakia; OLP, oral lichen planus; OSF, oral submucous fibrosis; UW, unstimulated whole; CE, capillary electrophoresis; GABA, γ-aminobutyric acid; GC, gas chromatography; LC, liquid chromatography analysis; QTOF-MS, quadrupole/time-of-flight mass spectrometry; HRMS, high-resolution mass spectrometry; UPLC, liquid chromatography; MS, mass spectrometry; partial least squares-discriminant ultra-performance liquid chromatography CPSI-MS, conductive polymer spray ionization mass spectrometry; CE-MS, capillary electrophoresis mass spectrometry; GC-MS, gas chromatography mass spectrometry; NMR, nuclear magnetic resonance; HPLC, high-performance liquid chromatography.

**Table 3:** Metabolic regulation of significant metabolites between oral potentially malignant disorder (OPMD) and oral squamous cell carcinoma (OSCC), OPMD vs. normal and OSCC vs. normal.

Metabolites	Sample Type	OPMD	OPMD vs. Normal	OPMD vs. OSCC	OSCC vs. Normal	References
Phenylalanine	Saliva	OLK	High	High	Low	Wei et al. [43]
Valine	Saliva	OLK		High		
n-eicosanoic acid	Saliva	OLK		Low		
Alanine	Saliva	OLK		Low		
n-tetradecanoic acid	Saliva	OLK		High		
n-dodecanoic acid	Saliva	OLK		High		
3-indoleproponoic acid	Saliva	OLK		Low		
Homocysteine	Saliva	OLK	High			
4-methoxyphenylacetic acid	Saliva	OLK	High			
Isoleucine	Saliva	OLK	Low	High	Low	
Lactic acid	Saliva	OLK	High	Low	High	Wei et al. [37]
	Tissue	OLP	High			Yang et al. [57]
Proline	Saliva	OLK	Low	High	Low	Wei et al. [43]
Threonine	Saliva	OLK	Low	High	Low	Musharraf et al. [60]
Glutamine	Serum	OLK	Low	High	Low	Gupta et al. [46]
	Tissue	OSF				Musharraf et al. [60]
	Tissue	OLP	High			Yang et al. [57]
Acetone	Serum	OLK		Low		Gupta et al. [46]
Acetate	Serum	OLK		Low		
Choline	Serum	OLK	High	Low	High	Gupta et al. [46]
						Kong et al. [37]
	Tissue	OLP	High			Yang et al. [57]
Ornithine	Saliva	OED	Low			Ishikawa et al. [50]
	Urine	OLP	High			Yang et al. [58]
Carnitine	Saliva	OED	Low			Ishikawa et al. [50]
o-hydroxybenzoate	Saliva	OED	Low			
n-acethylglucosamine-1-phosphate	Saliva	OED	Low			
Ribose-5-phosphate	Saliva	OED	Low			
Trimethylamine N-oxide	Saliva			Low		
Arginine	Saliva	OED	Low			Ishikawa et al. [50]
			High			Wang et al. [53]
Putrescine	Saliva	OLK	High	Low	High	Ishikawa et al. [63]
						Song et al. [65]
Creatinine	Saliva	OLK		Low		Ishikawa et al. [63]
5-aminovalerate	Saliva	OLK		Low		
Pipocolate	Saliva	OLK		Low		
N-acetylputrescine	Saliva	OLK		Low		
Gamma-butyrobetaine	Saliva	OLK		Low		
Indole 3-acetate	Saliva	OLK		Low		
N1-acetylspermine	Saliva	OLK		Low		
2′-deoxyinosine	Saliva	OLK		Low		
Ethanolamine phosphate	Saliva	OLK		Low		
N-acetylglucosamine	Saliva	OLK		Low		
N-acetylhistidine	Saliva	OLK		High		
o-acetylcarnitine	Saliva	OLK		High		
Glucose	Saliva	OLK	Low	High	Low	
Decanedioic acid,	Saliva	OLK		Low	HIgh	Tantray et al. [15]
2-methyloctacosane	Saliva	OLK		Low	HIgh	
octane	Saliva	OLK		Low	HIgh	
3,5-dimethyl	Saliva	OLK		Low	HIgh	
Pentadecane	Saliva	OLK		Low	HIgh	
Hentriacontane	Saliva	OLK		Low	HIgh	
Nonadecane	Saliva	OLK		Low	HIgh	
Oxalic acid	Saliva	OLK		Low	HIgh	
6-phenylundecanea	Saliva	OLK		Low	HIgh	
l-proline	Saliva	OLK		Low	HIgh	
2-furancarboxamide	Saliva	OLK		Low	HIgh	
2-isopropyl-5-methyl-1-heptanol	Saliva	OLK		Low	HIgh	
Pentanoic acid	Saliva	OLK		Low	HIgh	
Docosane	Saliva	OLK		Low	HIgh	
Eicosane	Saliva	OLK		High	Low	
2-methyl-4-keto-pentan-2-ol	Tissue	OSF	High	Low	High	Musharraf et al. [60]
(6E)-2,6-Dimethyl-2,6-octadiene	Tissue	OSF	Low	High		
3-heptanol	Tissue	OSF	Low	High	Low	
4-hydroxybenzaldehyde	Tissue	OSF	Low	High	Low	
cis-p-methan-3-one	Tissue	OSF	Low	High	Low	
Ethylene glycol	Tissue	OSF	Low	High	Low	
Geraniol formte	Tissue	OSF	Low	High	Low	
Glycine	Tissue	OSF	Low	High	Low	
Lysine	Tissue	OSF	Low	High	Low	
Melibiose	Tissue	OSF	High	Low	High	
Stearic acid	Tissue	OSF	High	Low	High	
Norleucine	Tissue	OSF	Low	High	Low	
Glutamic acid	Tissue	OSF	Low	High	Low	Musharraf et al. [60]
	Serum	OLP				Wang et al. [53]
	Tissue	OLP	High			Yang et al. [57]
Serine	Tissue	OSF	Low	High	Low	Musharraf et al. [60]
	Saliva	OLK				Song et al. [41]
	Tissue	OLP	High			Yang et al. [57]
Cadaverine	Saliva	OLK	High	Low	High	Song et al. [41]
Thymidine	Saliva	OLK	High	Low	High	
Adenosine	Saliva	OLK	High	Low	High	
5-aminopentoate	Saliva	OLK	High	Low	High	
Hippuric acid	Saliva	OLK	High	High	High	
Phospotocholine	Saliva	OLK	Low	High	Low	
Adrenic acid	Saliva	OLK	Low	High	Low	
6-hydroxynicotinic acid	Urine	OLK		High		Xie et al. [40]
Tyrosine	Urine	OLK		Low		
Cysteine	Urine	OLK		Low		
LysoPE(20:2)	Serum	OLP	High			Wang et al. [53]
LysoPE(20:4)	Serum	OLP	High			
LysoPC(20:4)	Serum	OLP	High			
3-Indoxyl sulphate	Serum	OLP	Low			
Testosterone sulfate	Serum	OLP	Low			
5-Aminopentanamide	Serum	OLP	Low			
11,12-EpETrE	Serum	OLP	Low			
Eicosapentaenoic acid	Serum	OLP	Low			
Sphingosine 1- phosphate	Serum	OLP	Low			
Pyroglutamic acid	Serum	OLP	Low			
LysoPC(16:1)	Serum	OLP	Low			
LysoPC(P-16:0)	Serum	OLP	Low			
LysoPC(P-16:0)	Serum	OLP	Low			
Citric acid	Serum	OLP	Low			
Acetyl-L-carnitine	Serum	OLP	Low			
Methionine	Serum	OLP	Low			
Leucine/Isoleucine/Norleucine	Serum	OLP	Low			
Uric acid	Serum	OLP	Low			
p-Chlorophenylalanine	Tissue	ROLP	Low			Yang et al. [57]
	Urine	ROLP	High			Yang et al. [58]
6,8-Dihydroxypurine	Tissue	ROLP	High			Yang et al. [57]
Malic acid	Tissue	ROLP	High			
Adrenochrome	Tissue	ROLP	Low			
Aspartic acid	Tissue	ROLP	High			
L-Acetylcartniitine	Tissue	ROLP	High			
Didymin	Tissue	ROLP	Low			
S-ethyl isothiourea	Tissue	ROLP	Low			
PE(P-18:1(9Z)/16:1(9Z)	Tissue	ROLP	Low			
Threonic acid	Tissue	ROLP	High			
L-histidine	Tissue	ROLP	High			
L-tryptophan	Tissue	ROLP	Low			
Prostaglandin E2	Tissue	ROLP	High			
Guanine	Tissue	ROLP	Low			
Taurine	Serum	OLP	Low			Wang et al. [53]
	Serum	OLP	Low			Sridharan et al. [61]
	Tissue	OLP	High			Yang et al. [57]
Citric acid	Serum	OLP	Low			Wang et al. [53];
	Tissue	OLP	High			Yang et al. [57]
Pro-Leu	Urine	ROLP	Low			Yang et al. [58]
Oxalacetic acid	Urine	ROLP	High			
Histidinol	Urine	ROLP	High			
Aminoacetone	Urine	ROLP	High			
N,N-Demetylaniline	Urine	ROLP	High			
14-cis-Retinal	Urine	ROLP	Low			
Kynurenic acid	Urine	ROLP	Low			
5-Aminopentanoic acid	Urine	ROLP	Low			
Isobutyryl carnitine	Urine	ROLP	Low			
Methylarsonite	Urine	ROLP	High			
3,4-Dihydroxymandelic acid	Urine	ROLP	High			
Chlorate	Urine	ROLP	High			
Hexadecanamide	Urine	ROLP	High			
Ala-His	Urine	ROLP	Low			
Succinic acid	Urine	ROLP	High			
Anthranilic acid	Urine	ROLP	Low			
Asn-Thr	Urine	ROLP	Low			
Methoxyacetic acid	Urine	ROLP	High			
D-(-)_Lyxose	Urine	ROLP	Low			
Carnosine	Urine	ROLP	Low			
Trp-Val	Urine	ROLP	Low			
Arg-Ile	Urine	ROLP	Low			
2-Oxo-4-methylthiobutanoic acid	Urine	ROLP	High			
Phe-Asp	Urine	ROLP	Low			
2-trans,4-cis-Decadienoylcarnitine	Urine	ROLP	Low			
9-Decenoylcarnitine	Urine	ROLP	Low			
Arg-Thr	Urine	ROLP	Low			
PE(20:5(5Z,8Z,11Z,14Z,17Z)/20:5(5Z,8Z,11Z,14Z,17Z))	Serum	EOLP	High			Yang et al. [56]
PE(14:0/14:0)	Serum	EOLP	High			
LysoPE(0:0/18:0)	Serum	EOLP	High			
Prostaglandin G1	Serum	EOLP	High			
Leukotriene D4	Serum	EOLP	High			
Protoporphyrinogen IX	Serum	EOLP	High			
VPGPR Enterostatin	Serum	EOLP	High			
Casomorphin	Serum	EOLP	High			
Pregnanediol	Serum	EOLP	Low			
20-Carboxy-leukotriene B4	Serum	EOLP	High			
Deoxycholic acid disulfate	Saliva	OLK	High		High	Sridharan et al. [42]
6-beta-hydroxytriamcinolone acetonide	Saliva	OLK	High		High	
Etoposide glucuronide	Saliva	OLK	High		High	
Sativic acid	Saliva	OLK	High		High	
13-cis-retinol	Saliva	OLK	High		High	
16-iodo-hexadecanoic acid	Saliva	OLK	High		High	
Prephytoene diphosphate	Saliva	OLK	High		High	
PGF1a alcohol	Saliva	OLK	High		High	
Hexadecanedioic acid	Saliva	OLK	High		High	
Tetradecanedioic acid	Saliva	OLK	High		High	
17beta-Estradiol	Saliva	OLK	High		High	
Hydroquinine 10,11-dihydroxy	Saliva	OLK	High		High	
1-methylhistidine	Saliva	OLK	High		High	
Inositiol 1,3,4-triphosphate	Saliva	OLK	High		High	
Pseudoridine	Saliva	OLK	High		High	
Spaglumic acid	Saliva	OLK	High		High	
D-Glycerate 2-phosphate	Saliva	OLK	High		High	
2-Hyroxymestranol	Saliva	OLK	High		High	
Octopine	Saliva	OLK	High		High	
Fumarylacetoacetic acid	Saliva	OLK	High		High	
4-Nitroquinoline -1 oxide	Saliva	OLK	High		High	
Estrone 3-sulfate	Saliva	OLK	High		High	
Etidronic acid	Saliva	OLK	High		High	
Dihydroisolysergic acid II	Saliva	OLK	High		High	
2-Oxoarginine	Saliva	OLK	High		High	
Octadecanoic acid	Saliva	OLK	High		High	
Norcocaine nitroxude	Saliva	OLK	High		High	
gamma-Aminobutry-lysine	Saliva	OLK	High		High	
9-choloro-10-hydroxy-hexa decanoic acid	Saliva	OLK	High		High	
Dextrophan sulfate	Saliva	OLK	High		High	
Sphinganine-1-phosphate	Saliva	OLK	High		High	
4-Hydroaminoquinolne N-oxide	Saliva	OLK	High		High	
Betana	Saliva	OLK	High		High	
Undecaprenyl diphosphate	Saliva	OLK	High		High	
D-Urobillinogen	Saliva	OLK	High		High	
Estrone-3-glucoronide	Saliva	OLK	High		High	
(S)-Ureidoglycolic acid	Saliva	OLK	High		High	
12-amino-octadecanoic acid	Saliva	OLK	Low		Low	
Ubiquinone	Saliva	OLK	Low		Low	
Deoxypodophyllotoxin	Saliva	OLK	Low		Low	
Zolpidem Metabolite II	Saliva	OLK	Low		Low	
Estradiol Valerate	Saliva	OLK	Low		Low	
Neuraminic acid	Saliva	OLK	Low		Low	
L-Homocysteic acid	Saliva	OLK	Low		Low	
Isosorbide dinitrate	Saliva	OLK	Low		Low	
Muramic acid	Saliva	OLK	Low		Low	
Retinol phosphate	Saliva	OLK	Low		Low	
3-Hydroxylidocaine glucuronide	Saliva	OLK	Low		Low	
Citrate	Cell line	OLK	High			
Homocysteine	Cell line	OLK	High			Karen-Ng et al. [48]
N1-methyladenosine	Cell line	OLK	High			
Glutathione	Cell line	OLK	High			
Gulono-1,4-lactone	Cell line	OLK	High			
Glutamate	Cell line	OLK	Low			
Beta-hydroxyisovalerate	Cell line	OLK	Low			
Alpha-hydroxyisovalerate	Cell line	OLK	Low			
3-hydroxyisobutyrate	Cell line	OLK	Low			
Myristoleate	Cell line	OLK	Low			
Palmitoleate	Cell line	OLK	Low			
10-heptadecenoate	Cell line	OLK	Low			
Docosapentaenoate	Cell line	OLK	Low			
Docosahexaenoate	Cell line	OLK	Low			
Linoleate	Cell line	OLK	Low			
Arachidonate	Cell line	OLK	Low			
Glycerol	Cell line	OLK	Low			
Hypoxanthine	Cell line	OLK	Low			Karen-Ng et al. [48];
	Serum	OLP	Low			Wang et al. [53]

OED, oral epithelial dysplasia; OLK, oral leukoplakia; OLP, oral lichen planus; OSF, oral submucous fibrosis; OSCC, oral squamous cell carcinoma; ROLP, reticular oral lichen planus; EOLP, erosive oral lichen planus.

## 5. Clinical Utility of Using Metabolomics for Oral Leukoplakia

Cancer cells demonstrate an elevated rate of nutrient consumption and rerouting metabolic processes to favor de novo biosynthesis. Targeting altered metabolic pathways may provide novel therapeutic approaches in cancer research [66].

**Figure 2:**
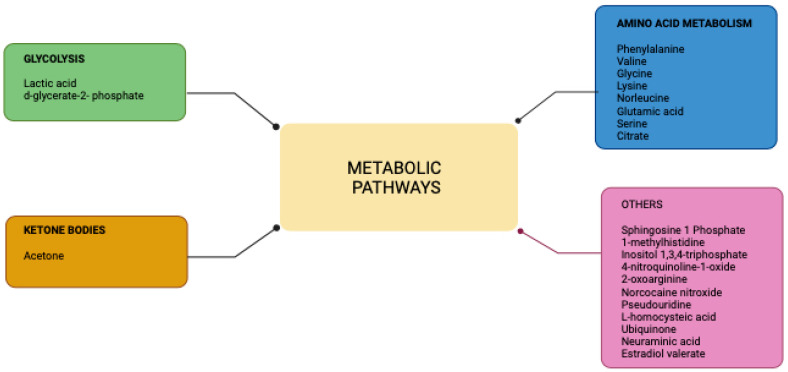
This diagram displays the metabolomic pathways and their related metabolites associated with oral leukoplakia.

In this section, we discuss studies describing how metabolomics aids in the discovery of new targets for therapy. We narrowed down our main focus of OPMD to oral leukoplakia (OLK) due to its highest rate of transforming into oral malignancy. Table 4 shows several amino acids that were considered to be important differential amino acids between OLK and OSCC and OLK and normal oral mucosa from different sample types, which could be regarded as potential biomarkers in OLK. We grouped these metabolites into respective metabolic pathways (Figure 2). The majority of the metabolites involved in amino acid metabolism and glycolysis.

Metabolomic reprogramming is one of the hallmarks of cancer. Metabolomic changes in cancer involve various aspects of metabolism, including glucose deregulation, the uptake and consumption of amino acids, and the application of glycolysis/tricarboxylic acid (TCA) cycle intermediates in biosynthesis. Glycolysis is a basic metabolic pathway of carbohydrates that converts glucose into pyruvates. The goal of glycolysis is to provide energy and intermediates for other metabolic pathways in the form of ATP and reduced equivalents of NADH [67]. In normal cells, glucose is converted into pyruvate in the presence of oxygen and into lactate in the absence of or limited oxygen [68]. However, in cancer cells, glucose is converted into lactate even in the presence of sufficient oxygen. Decreased glucose and increased lactate levels, which indicate ongoing glycolysis, are known as the Warburg effect (or aerobic glycolysis), which also contributes to cancer survival and growth [41].

Numerous studies have investigated metabolic changes in the early stage of cancer development and reported some preliminary signs of metabolic reprogramming in premalignant cells and tissues. For instance, an early imaging study revealed elevated glycolysis and glutamine consumption in precancerous epithelial tissues [69]. Significantly high levels of metabolism-related genes were seen in precancerous colorectal lesions compared to normal tissues [70]. A rat model study of early-stage hepatocarcinogenesis also observed a shift from oxidative phosphorylation to the Warburg effect [71]. A recent study by Xun Chen et al. [49] revealed an increase in the level of pyruvate in oral premalignant cells, as well as glucose uptake and higher lactate production in cervical premalignant lesions. These findings support metabolomic reprogramming to the Warburg effect in premalignant lesions. The study also found glutamine, lysine, and methionine to be differentially expressed between dysplastic oral keratinocytes (DOK) and human oral epithelial cells (HOEC), which suggested amino acid metabolism could potentially play a crucial role in the progression of oral premalignant lesions. Similarly, Gupta et al. [46] and Musharaff et al. [60] also reported glutamine to be differentially expressed between OPMD and OSCC.

A decrease in amino acid levels was shown to be associated with increased energy metabolism or the upregulation of related biosynthesis pathways, which are necessary for cancer cell proliferation. For instance, glutamine levels are low in cancer patients, as it is used by cancer cells to produce energy and for biosynthetic purposes. It may be linked to amino acid catabolism and the suppression of the Krebs cycle. Targeting amino acid metabolic enzymes unveils a promising strategy for cancer therapy, particularly for the development of novel therapeutic agents. Currently, several drugs are undergoing clinical trials that specifically target amino acid metabolic pathways in cancer cells [72]. However, we noted that the metabolic changes of OPMD are varied depending on the type of samples. For instance, Gupta et al. (2015) [46] reported a decrease in glutamine in serum samples of diseased conditions compared to the healthy controls, but Xun et al. [49] reported an increase in glutamine in diseased conditioned cell lines compared to the controls. This was also seen in the levels of phenylalanine, valine, and lactate, which change differently in OLK serum and saliva samples. This is probably due to the different metabolic rates in the different biological compartments, which changes differently in response to pathophysiological stimuli [43,73]. The significant upregulation of 2-phospho-D-glyceric acid suggests that glycolysis is highly utilized during cell proliferation in OSCC, even in the presence of oxygen [43]. Whereas the downregulation of amino acids is associated with increased glycolysis during cell proliferation in cancer cells. This could be due to its utilization by cancer cells to meet the increased energy demand [74].

Fatty acids are also required by cancer cells for membrane synthesis and the production of lipid signaling molecules to promote cancer cell growth. For instance, acetone and acetate, which are at high levels in OSCC, showed that the rate of fatty acid metabolism is altered in diseased conditions. High levels of lactic acid, which is the end product of glycolysis, are also seen in OSCC compared to OLK and normal samples [43]. This suggests that cancer cells can switch the active TCA cycle to glycolysis and fatty acid oxidation as a backup mechanism by which to produce energy. Therefore, cancer cells are dependent on glycolysis and lipolysis as a main source of energy [46].

Metabolomic analysis also unveiled that citrate uptake could affect cancer cell metabolism via citrate-dependent metabolic pathways. Citrate is the primary substrate for fatty acid synthesis and is also involved in amino acid synthesis, which is crucial for proliferating cells. Interestingly, it has also been reported that high citrate levels released by senescent fibroblasts may be an extra source of these metabolites for cancer cells in vivo [75,76]. Several malignancies have been reported to change in the expression and/or location of pmCiC, the plasma membrane citrate transporter, and may be associated with the uptake of citrate and increased cancer growth and metastasis [76,77]. Intriguingly, a metabolomic study by Tiziani et al. [30] reported the depletion of citrate levels in sera from head and neck patients, leading to the development and progression of OSCC [30].

## 6. Future Direction

The identification of molecular markers is required to predict the risk of malignant progression in OPMDs. Clinical presentation and biopsy followed by histological examination remain the gold standard by which to predict the prognosis of OPMD and OSCC that are solely based on clinical and microscopic features with some degree of subjectivity.

The current approach to treating OPMD is largely a “wait and see” strategy, which is far from ideal. However, the potential for a more proactive approach lies in the identification and validation of specific biomarkers or metabolites that may also serve as therapeutic targets. Metabolic abnormalities often lead to the dysfunction of metabolic pathways and the accumulation or deficiency of certain metabolites, which are recognized hallmarks [78]. Therefore, the identification of these metabolites could provide valuable insights into the underlying mechanisms of OPMD and offer clinical utility as biomarkers of malignant progression and/or potential therapeutic targets.

The levels of alanine and glutamate are higher in OPMD compared to OSCC (Table 2). Metformin is commonly prescribed as an oral anti-diabetic drug for type 2 diabetes mellitus (T2DM) treatment [79] and has been studied intensively for its anti-neoplastic effects since it was approved by the Food and Drug Administration (FDA) in 1994. Substantial research has been linked to metformin to reduce mortality rates in colorectal, liver, esophageal, and stomach cancers [80]. One study also reported that metformin inhibits tumorigenesis by inhibiting glutaminase [81], and prolonged treatment with metformin decreased L-glutamate accumulation in MDA-MB-231 breast cancer cell lines [81]. Recently, a study by Glutkind et al. conducted a phase IIa clinical trial in OLK and oral erythroplakia to assess metformin’s ability to target P13K/mTOR signaling in preventing malignant transformation [82]. Based on these findings, we hypothesize that the use of metformin for OPMD patients could target glutaminase, potentially reducing the risk of progression to cancer.

Glutamate plays a crucial role in cancer metabolism by serving as a source of energy for cancer cells through ATP production. Glutamate was also found to be regulated via glutaminase together with ALDH, contributing to cancer stemness [83]. The role of alanine in cancer is less understood, but emerging evidence suggests that alanine biosynthesis is associated with proliferative cell metabolism. In pancreatic cancer, the secretion of alanine can be utilized by the TCA cycle of the cancer cells [84]. Further research is needed to fully understand these complex metabolic interactions and their implications for cancer treatment strategies.

Mycielska M.E et al. (2019) conducted a study demonstrating the role of gluconate in cancer immunotherapy, highlighting its emerging potential as an anti-cancer mediator. Despite there being only a limited number of studies and trials involving gluconate, its potential as an anti-cancer mediator is becoming increasingly apparent. For instance, Zn^2+^ gluconate, when used as an adjuvant therapy, significantly enhanced the efficacy of standard chemotherapy in treating children with acute lymphocytic leukaemia [85]. Conversely, a separate study reported that cisplatin, when administered without gluconate as the adjuvant therapy, showed no improvement [86]. Recently, it was reported that gluconate could target citrate by inhibiting the citrate transporter (pmCiC) in cancer cells and abrogate tumor growth in mice [86]. These discoveries present an interesting hypothesis, that gluconate could be a novel diagnostic and treatment option in cancer research. In OPMD, elevated levels of citrate have been observed compared to normal counterparts. Thus, targeting citrate with gluconate could be a potential therapeutic strategy for OPMD. Taken together, these studies strongly suggest that targeting metabolites at the early stage of disease might be a promising strategy for targeted therapy in oral cancer.

There are still a number of limitations in using metabolomic profiling to distinguish OPMD or OSCC. It is impossible to achieve a complete homogeneity across different batches of biological samples. Therefore, the methods and techniques used should be optimized to establish highly sensitive routine procedures that can be applied to metabolites with different polarities across different samples. Studies to include OPMDs with various rates of malignant transformation would also be beneficial to identify biomarkers of MT. However, this would be challenging because some lesions, such as erythroplakia, are very uncommon, or in some cases, such as proliferative verrucous leukoplakia, necessitate prolonged follow-up to establish a definitive status [87]. Furthermore, developing therapies that specifically target glucose metabolism enzymes also continues to pose a challenge. The presence of multiple isoforms of glucose metabolism enzymes with similar structures results in low selectivity in targeted drugs and the compensatory activation of other isoforms in tumors. Their poor targeting may also lead to harmful side effects, which presents difficulties in fulfilling the requirements for cancer treatment [88,89,90].

## 7. Conclusions

Metabolomic profiling studies are able to identify potential biomarker metabolites that distinguish OPMD from OSCC and control groups. In fact, certain metabolites can even effectively differentiate between high-risk and low-risk oral epithelial dysplasia, highlighting their potential in identifying progressive versus non-progressive OPMDs. This suggests that these metabolites could be valuable biomarkers for stratifying OPMDs, aiding in early intervention and tailored treatment approaches. However, caution is needed to validate and optimize these metabolites before they can be used clinically, but they may pave the way for the development of non-invasive diagnostic tools and personalized therapeutic approaches for OPMD and OSCC.

## Figures and Tables

**Table 1:** The clinical appearance of oral potentially malignant disorders (OPMDs).

OPMD Entity	Clinical Appearance
Oral leukoplakia	White patches or lesions, may have a rough, thickened, or fissured surface
Oral erythroplakia	Red, velvety lesions that may be flat or slightly raised appearance
Oral erythroleukoplakia	Mixed white-and-red lesions with atrophy or specked presentation
Proliferative verrucous leukoplakia	Multifocal white plaques often with a verrucous, keratotic surface
Oral submucous fibrosis	Pale, marble-like patches, and progressive stiffening of oral mucosa, leading to restricted mouth opening
Oral lichen planus	Usually white, reticular, and plaque-like, with atrophic or erosive presentation
Oral lichenoid lesions	White-and-red lesions with a reticular, striated presentation

**Table 4:** Potential malignant transformation biomarkers associated with oral leukoplakia (OLK).

Metabolites	OPMD vs. OSCC	OPMD vs. Normal
Phenylalanine	High	High
Valine	High	
Glutamine	High	Low
Lactic acid	Low	
Acetone	Low	
Acetate	Low	
Choline	Low	
Serine	Low	
Citrate		High
1-methylhistidine		High
Inositol 1,3,4-triphosphate		High
d-glycerate-2-phosphate		High
4-nitroquinoline-1-oxide		High
2-oxoarginine		High
Norcocaine nitroxide		High
Sphinganine-1-phosphate		High
Pseudouridine		High
L-homocysteic acid		Low
Ubiquinone		Low
Neuraminic acid		Low
Estradiol valerate		Low

## Data Availability

The data that supports the findings of this study are available from the corresponding authors upon reasonable request.
